# Supplementary material for: Using Maize δ15N values to assess soil fertility in fifteenth- and sixteenth-century ad Iroquoian agricultural fields
Source: PLoS One. 2020 Apr 8;15(4):e0230952. doi: 10.1371/journal.pone.0230952 (PMC7141618; doi:10.1371/journal.pone.0230952)
Supplement: S1 Table — (DOCX) [file pone.0230952.s001.docx]

Table S1. Stable isotope results of maize kernel charring experiments.

| δ^15^N | time (hr) | raw | 180°C | 220°C | 260°C | Δ | δ^13^C | time (hr) | raw | 180°C | 220°C | 260°C | Δ |
| --- | --- | --- | --- | --- | --- | --- | --- | --- | --- | --- | --- | --- | --- |
| 1Y | 2 | 0.74 | 0.66 |  |  | -0.08 | 1Y | 2 | -12.15 | -12.13 |  |  | -0.02 |
| 2Y | 2 | 0.52 | 0.67 |  |  | 0.15 | 2Y | 2 | -12.01 | -12.08 |  |  | 0.07 |
| 13W | 2 | 2.57 | 2.89 |  |  | 0.32 | 13W | 2 | -11.49 | -11.41 |  |  | -0.08 |
| 14W | 2 | 2.51 | 2.77 |  |  | 0.26 | 14W | 2 | -11.34 | -11.34 |  |  | 0.00 |
| 19H | 2 | 4.78 | 4.73 |  |  | -0.05 | 19H | 2 | -10.70 | -10.59 |  |  | -0.11 |
| 20H | 2 | -0.02 | 0.07 |  |  | 0.09 | 20H | 2 | -11.20 | -10.74 |  |  | -0.46 |
| 28Y | 2 | -0.2 | 0.25 |  |  | 0.45 | 28Y | 2 | -11.55 | -11.99 |  |  | 0.44 |
| 29Y | 2 | -0.16 | 0.16 |  |  | 0.32 | 29Y | 2 | -11.95 | -11.89 |  |  | -0.06 |
| 30Y | 2 | 0.55 | 0.09 |  |  | -0.46 | 30Y | 2 | -11.58 | -12.11 |  |  | 0.53 |
| 31Y | 2 | -1 | -1.78 |  |  | -0.78 | 31Y | 2 | -11.73 | -11.44 |  |  | -0.29 |
| 32Y | 2 | 0.58 | 0.99 |  |  | 0.41 | 32Y | 2 | -11.73 | -12.06 |  |  | 0.33 |
| 38W | 2 | 2.45 | 2.28 |  |  | -0.17 | 38W | 2 | -10.81 | -10.81 |  |  | 0.00 |
| 39W | 2 | 2.41 | 2.72 |  |  | 0.31 | 39W | 2 | -10.90 | -10.71 |  |  | -0.19 |
| 40W | 2 | 2.35 | 1.9 |  |  | -0.45 | 40W | 2 | -11.32 | -11.34 |  |  | 0.02 |
| 41W | 2 | 2.17 | 2.4 |  |  | 0.23 | 41W | 2 | -11.07 | -11.32 |  |  | 0.25 |
| 42W | 2 | 1.96 | 2.46 |  |  | 0.50 | 42W | 2 | -11.40 | -11.35 |  |  | -0.05 |
| 48H | 2 | 0.53 | -0.21 |  |  | -0.74 | 43W | 2 | -11.26 | -11.06 |  |  | -0.20 |
| 49H | 2 | 5.86 | 4.67 |  |  | -1.19 | 44W | 2 | -10.76 | -11.03 |  |  | 0.27 |
| 50H | 2 | 4.44 | 5.42 |  |  | 0.98 | 48H | 2 | -11.16 | -11.06 |  |  | -0.10 |
| 52H | 2 | 6.56 | 7.70 |  |  | 1.14 | 49H | 2 | -11.22 | -10.40 |  |  | -0.82 |
| 1903Y-U | 24 | -1.41 | -0.17 |  |  | 1.24 | 50H | 2 | -10.63 | -10.88 |  |  | 0.25 |
| 1905Y-U | 24 | 1.16 | 1.26 |  |  | 0.10 | 51H | 2 | -10.39 | -10.51 |  |  | 0.12 |
| 1904Y-U | 24 | -1.44 | -0.83 |  |  | 0.61 | 52H | 2 | -10.76 | -10.77 |  |  | 0.01 |
| 1908W-U | 24 | 2.23 | 2.81 |  |  | 0.58 | 1903Y-U | 24 | -12.00 | -12.32 |  |  | 0.32 |
| 1909W-U | 24 | 3.25 | 2.48 |  |  | -0.77 | 1905Y-U | 24 | -11.86 | -12.07 |  |  | 0.21 |
| 1910W-U | 24 | 2.08 | 2.66 |  |  | 0.58 | 1904Y-U | 24 | -12.17 | -12.03 |  |  | -0.14 |
| 1915H-U | 24 | 0.64 | 0.14 |  |  | -0.50 | 1908W-U | 24 | -10.94 | -11.13 |  |  | 0.19 |
| 1914H-U | 24 | 0.83 | 1.62 |  |  | 0.79 | 1909W-U | 24 | -11.49 | -11.29 |  |  | -0.20 |
| 1913H-U | 24 | 3.71 | 4.63 |  |  | 0.92 | 1910W-U | 24 | -11.32 | -11.14 |  |  | -0.18 |
| 3Y | 2 | 0.54 |  | 0.70 |  | 0.16 | 1915H-U | 24 | -11.42 | -10.59 |  |  | -0.83 |
| 4Y | 2 | 0.75 |  | 0.85 |  | 0.10 | 1914H-U | 24 | -11.04 | -11.03 |  |  | -0.01 |
| 10W | 2 | 2.53 |  | 2.94 |  | 0.41 | 1913H-U | 24 | -10.73 | -10.73 |  |  | 0.00 |
| 11W | 2 | 2.64 |  | 2.88 |  | 0.24 | 10W | 2 | -10.67 |  | -11.00 |  | 0.33 |
| 21H | 2 | 1.32 |  | 2.47 |  | 1.15 | 11W | 2 | -11.06 |  | -11.13 |  | 0.07 |
| 22H | 2 | 5.30 |  | 5.75 |  | 0.45 | 3Y | 2 | -12.19 |  | -12.11 |  | -0.08 |
| 34Y | 2 | -1.88 |  | -0.96 |  | 0.92 | 4Y | 2 | -12.06 |  | -12.25 |  | 0.19 |
| 36Y | 2 | -0.82 |  | 0.89 |  | 1.71 | 21H | 2 | -10.81 |  | -11.00 |  | 0.19 |
| 37Y | 2 | 0.63 |  | 1.17 |  | 0.54 | 22H | 2 | -11.41 |  | -11.53 |  | 0.12 |
| 43W | 2 | 2.52 |  | 2.55 |  | 0.03 | 36Y | 2 | -11.61 |  | -12.09 |  | 0.48 |
| 44W | 2 | 2.67 |  | 2.54 |  | -0.13 | 37Y | 2 | -12.13 |  | -12.25 |  | 0.12 |
| 45W | 2 | 2.42 |  | 2.49 |  | 0.07 | 45W | 2 | -11.14 |  | -10.84 |  | -0.30 |
| 46W | 2 | 2.29 |  | 2.51 |  | 0.22 | 46W | 2 | -11.35 |  | -11.04 |  | -0.31 |
| 47W | 2 | 2.11 |  | 2.79 |  | 0.68 | 47W | 2 | -11.94 |  | -12.15 |  | 0.21 |
| 51H | 2 | 3.63 |  | 3.81 |  | 0.18 | 54H | 2 | -10.97 |  | -10.25 |  | -0.72 |
| 53H | 2 | 0.73 |  | 2.07 |  | 1.34 | 55H | 2 | -10.86 |  | -10.23 |  | -0.63 |
| 54H | 2 | 6.35 |  | 5.70 |  | -0.65 | 56H | 2 | -10.69 |  | -11.01 |  | 0.32 |
| 55H | 2 | 0.77 |  | 1.57 |  | 0.80 | 57H | 2 | -10.50 |  | -10.77 |  | 0.27 |
| 56H | 2 | 4.55 |  | 6.04 |  | 1.49 | 1920Y-U | 24 | -12.04 |  | -11.9 |  | -0.11 |
| 57H | 2 | 9.87 |  | 10.31 |  | 0.44 | 1919Y-U | 24 | -12.45 |  | -12 |  | -0.48 |
| 1920Y-U | 24 | 0.48 |  | 1.04 |  | 0.56 | 1918Y-U | 24 | -11.85 |  | -12 |  | 0.14 |
| 1919Y-U | 24 | 1.8 |  | 1.63 |  | -0.17 | 1925W-U | 24 | -11.28 |  | -11.2 |  | -0.10 |
| 1918Y-U | 24 | 0.12 |  | 0.88 |  | 0.76 | 1923W-U | 24 | -10.71 |  | -10.8 |  | 0.07 |
| 1925W-U | 24 | 2.56 |  | 3.47 |  | 0.91 | 1924W-U | 24 | -11.18 |  | -11.2 |  | 0.04 |
| 1923W-U | 24 | 2.68 |  | 3.35 |  | 0.67 | 1928H-U | 24 | -10.90 |  | -10.6 |  | -0.26 |
| 1924W-U | 24 | 2.19 |  | 3.18 |  | 0.99 | 1930H-U | 24 | -10.91 |  | -10.3 |  | -0.63 |
| 1928H-U | 24 | 2.57 |  | 3.31 |  | 0.74 | 1929H-U | 24 | -10.87 |  | -10.8 |  | -0.10 |
| 1930H-U | 24 | 5.87 |  | 5.85 |  | -0.02 | 12W | 2 | -11.27 |  |  | -12.38 | 1.11 |
| 1929H-U | 24 | 0.43 |  | 1.41 |  | 0.98 | 23H | 2 | -10.86 |  |  | -11.60 | 0.74 |
| 5Y | 2 | 1.21 |  |  | 2.03 | 0.82 | 24H |  | -11.21 |  |  | -11.79 | 0.58 |
| 6Y | 2 | 0.17 |  |  | 1.10 | 0.93 | 15W |  | -11.32 |  |  | -11.97 | 0.65 |
| 12W | 2 | 2.50 |  |  | 3.32 | 0.82 | 5Y |  | -12.11 |  |  | -12.39 | 0.28 |
| 15W | 2 | 2.54 |  |  | 3.42 | 0.88 | 6Y |  | -12.17 |  |  | -12.19 | 0.02 |
| 23H | 2 | 6.33 |  |  | 7.69 | 1.36 |  |  |  |  |  |  |  |
| 24H | 2 | 7.08 |  |  | 8.03 | 0.95 |  |  |  |  |  |  |  |
