# Supplementary material for: Using Maize δ15N values to assess soil fertility in fifteenth- and sixteenth-century ad Iroquoian agricultural fields
Source: PLoS One. 2020 Apr 8;15(4):e0230952. doi: 10.1371/journal.pone.0230952 (PMC7141618; doi:10.1371/journal.pone.0230952)
Supplement: S2 Table — (DOCX) [file pone.0230952.s002.docx]

Table S2. Herbivore bone collagen δ15N values used to calculate forage values.

| **Sample** | **Species** | **δ^15^N** | **Location** | **Reference** |
| --- | --- | --- | --- | --- |
| DOR16.1 | deer *(Odocoileus virginianus)* | 5.44 | Ontario | Booth (2014) |
| DOR17.1 | deer *(Odocoileus virginianus)* | 6.06 | Ontario | Booth (2014) |
| DOR18.1 | deer *(Odocoileus virginianus)* | 4.9 | Ontario | Booth (2014) |
| DOR19.1 | deer *(Odocoileus virginianus)* | 5.86 | Ontario | Booth (2014) |
| HOL03.1 | deer *(Odocoileus virginianus)* | 6.12 | Ontario | Booth (2014) |
| HOL15.1 | deer *(Odocoileus virginianus)* | 4.62 | Ontario | Booth (2014) |
| HOL23.1 | deer *(Odocoileus virginianus)* | 6.35 | Ontario | Booth (2014) |
| HOL24.1 | deer *(Odocoileus virginianus)* | 5.46 | Ontario | Booth (2014) |
| MCK03.1 | deer *(Odocoileus virginianus)* | 6.63 | Ontario | Booth (2014) |
| MCK04.1 | deer *(Odocoileus virginianus)* | 4.83 | Ontario | Booth (2014) |
| MCK12.1 | deer *(Odocoileus virginianus)* | 5.08 | Ontario | Booth (2014) |
| MCK13.1 | deer *(Odocoileus virginianus)* | 4.82 | Ontario | Booth (2014) |
| MCK17.1 | deer *(Odocoileus virginianus)* | 4.77 | Ontario | Booth (2014) |
| MCK19.1 | deer *(Odocoileus virginianus)* | 6.21 | Ontario | Booth (2014) |
| MCK20.1 | deer *(Odocoileus virginianus)* | 3.92 | Ontario | Booth (2014) |
| MCK21.1 | deer *(Odocoileus virginianus)* | 3.74 | Ontario | Booth (2014) |
| MCK22.1 | deer *(Odocoileus virginianus)* | 4.3 | Ontario | Booth (2014) |
| MCK23.1 | deer *(Odocoileus virginianus)* | 5.11 | Ontario | Booth (2014) |
| MCK33.1 | deer *(Odocoileus virginianus)* | 5.75 | Ontario | Booth (2014) |
| MCK46.1 | deer *(Odocoileus virginianus)* | 6.49 | Ontario | Booth (2014) |
| BrB-010 | deer *(Odocoileus virginianus)* | 4.75 | Ontario | Morris (2015) |
| BrB-011 | deer *(Odocoileus virginianus)* | 4.88 | Ontario | Morris (2015) |
| BrB-012 | deer *(Odocoileus virginianus)* | 4.35 | Ontario | Morris (2015) |
| BrB-013 | deer *(Odocoileus virginianus)* | 4.99 | Ontario | Morris (2015) |
| Bog-054 | deer *(Odocoileus virginianus)* | 5.03 | Ontario | Morris (2015) |
| Clv-015 | deer *(Odocoileus virginianus)* | 6.12 | Ontario | Morris (2015) |
| Clv-016 | deer *(Odocoileus virginianus)* | 5.68 | Ontario | Morris (2015) |
| Clv-017 | deer *(Odocoileus virginianus)* | 8.16 | Ontario | Morris (2015) |
| Clv-019 | deer *(Odocoileus virginianus)* | 5.13 | Ontario | Morris (2015) |
| Cra-001 | deer *(Odocoileus virginianus)* | 4.58 | Ontario | Morris (2015) |
| Crf-002 | deer *(Odocoileus virginianus)* | 4.15 | Ontario | Morris (2015) |
| Crf-095 | deer *(Odocoileus virginianus)* | 5.17 | Ontario | Morris (2015) |
| Dav-001 | deer *(Odocoileus virginianus)* | 3.9 | Ontario | Morris (2015) |
| Dav-003 | deer *(Odocoileus virginianus)* | 4.27 | Ontario | Morris (2015) |
| Dav-004 | deer *(Odocoileus virginianus)* | 6.16 | Ontario | Morris (2015) |
| Fon-001 | deer *(Odocoileus virginianus)* | 4.98 | Ontario | Morris (2015) |
| Fon-009 | deer *(Odocoileus virginianus)* | 6.14 | Ontario | Morris (2015) |
| Fon-014 | deer *(Odocoileus virginianus)* | 5.81 | Ontario | Morris (2015) |
| Fon-019 | deer *(Odocoileus virginianus)* | 5.46 | Ontario | Morris (2015) |
| Fon-019 mDUP | deer *(Odocoileus virginianus)* | 5.61 | Ontario | Morris (2015) |
| Fon-047 | deer *(Odocoileus virginianus)* | 5.12 | Ontario | Morris (2015) |
| Fon-047 DUP | deer *(Odocoileus virginianus)* | 2.83 | Ontario | Morris (2015) |
| Ham-004 | deer *(Odocoileus virginianus)* | 4.98 | Ontario | Morris (2015) |
| IWP(01)-001 | deer *(Odocoileus virginianus)* | 5.29 | Ontario | Morris (2015) |
| IWP(01)-001 mDUP | deer *(Odocoileus virginianus)* | 5.31 | Ontario | Morris (2015) |
| IWP(01)-009 | deer *(Odocoileus virginianus)* | 5.68 | Ontario | Morris (2015) |
| IWP(01)-025 | deer *(Odocoileus virginianus)* | 5.65 | Ontario | Morris (2015) |
| IWP(01)-025 DUP | deer *(Odocoileus virginianus)* | 5.34 | Ontario | Morris (2015) |
| IWP(01)-036 DUP | deer *(Odocoileus virginianus)* | 5.47 | Ontario | Morris (2015) |
| IWP(01)-036 mDUP | deer *(Odocoileus virginianus)* | 4.96 | Ontario | Morris (2015) |
| IWP(03)-23 | deer *(Odocoileus virginianus)* | 4.85 | Ontario | Morris (2015) |
| IWP(09)-002 | deer *(Odocoileus virginianus)* | 4.92 | Ontario | Morris (2015) |
| IWP(09)-047 | deer *(Odocoileus virginianus)* | 4.78 | Ontario | Morris (2015) |
| IWP(09)-047 DUP | deer *(Odocoileus virginianus)* | 4.8 | Ontario | Morris (2015) |
| IWP(09)-047 DUP | deer *(Odocoileus virginianus)* | 4.91 | Ontario | Morris (2015) |
| IWP(09)-054 | deer *(Odocoileus virginianus)* | 5.07 | Ontario | Morris (2015) |
| IWP(09)-054 mDUP | deer *(Odocoileus virginianus)* | 5.25 | Ontario | Morris (2015) |
| IWP(09)-134 | deer *(Odocoileus virginianus)* | 5.19 | Ontario | Morris (2015) |
| IWP(09)-134 mDUP | deer *(Odocoileus virginianus)* | 5.13 | Ontario | Morris (2015) |
| IWP(12)-003 | deer *(Odocoileus virginianus)* | 4.7 | Ontario | Morris (2015) |
| IWP(12)-004 | deer *(Odocoileus virginianus)* | 5.35 | Ontario | Morris (2015) |
| IWP(12)-005 | deer *(Odocoileus virginianus)* | 5.14 | Ontario | Morris (2015) |
| Lia-006 | deer *(Odocoileus virginianus)* | 6.82 | Ontario | Morris (2015) |
| Lia-010 | deer *(Odocoileus virginianus)* | 8.62 | Ontario | Morris (2015) |
| Mon-004 | deer *(Odocoileus virginianus)* | 5.96 | Ontario | Morris (2015) |
| Mon-005 | deer *(Odocoileus virginianus)* | 4.4 | Ontario | Morris (2015) |
| Mon-006 | deer *(Odocoileus virginianus)* | 4.78 | Ontario | Morris (2015) |
| Mon-007 | deer *(Odocoileus virginianus)* | 5.33 | Ontario | Morris (2015) |
| Mon-008 | deer *(Odocoileus virginianus)* | 5.41 | Ontario | Morris (2015) |
| OLG-001 | deer *(Odocoileus virginianus)* | 7 | Ontario | Morris (2015) |
| OLG-002 | deer *(Odocoileus virginianus)* | 6.07 | Ontario | Morris (2015) |
| OLG-013 | deer *(Odocoileus virginianus)* | 5.08 | Ontario | Morris (2015) |
| Pip(1)-103 | deer *(Odocoileus virginianus)* | 3.73 | Ontario | Morris (2015) |
| Pip(1)-157 | deer *(Odocoileus virginianus)* | 4.54 | Ontario | Morris (2015) |
| Por-009 | deer *(Odocoileus virginianus)* | 6.12 | Ontario | Morris (2015) |
| Por-017 | deer *(Odocoileus virginianus)* | 5.01 | Ontario | Morris (2015) |
| Por-017 mDUP | deer *(Odocoileus virginianus)* | 5.04 | Ontario | Morris (2015) |
| Pri-008 | deer *(Odocoileus virginianus)* | 4.95 | Ontario | Morris (2015) |
| Pri-017 | deer *(Odocoileus virginianus)* | 4.91 | Ontario | Morris (2015) |
| Pri-017 DUP | deer *(Odocoileus virginianus)* | 5.85 | Ontario | Morris (2015) |
| Pri-019 | deer *(Odocoileus virginianus)* | 4.41 | Ontario | Morris (2015) |
| Pri-019 DUP | deer *(Odocoileus virginianus)* | 2.99 | Ontario | Morris (2015) |
| Rif-007 | deer *(Odocoileus virginianus)* | 8.23 | Ontario | Morris (2015) |
| Rif-007 mDUP | deer *(Odocoileus virginianus)* | 8.05 | Ontario | Morris (2015) |
| Rif-077 | deer *(Odocoileus virginianus)* | 6.8 | Ontario | Morris (2015) |
| Sil-019 | deer *(Odocoileus virginianus)* | 5.54 | Ontario | Morris (2015) |
| Sil-019 DUP | deer *(Odocoileus virginianus)* | 5.48 | Ontario | Morris (2015) |
| Sil-026 | deer *(Odocoileus virginianus)* | 6.09 | Ontario | Morris (2015) |
| Sil-026 DUP | deer *(Odocoileus virginianus)* | 6.17 | Ontario | Morris (2015) |
| Sla-017 | deer *(Odocoileus virginianus)* | 5.82 | Ontario | Morris (2015) |
| Tho-002 | deer *(Odocoileus virginianus)* | 5.73 | Ontario | Morris (2015) |
| Tho-012 | deer *(Odocoileus virginianus)* | 5.75 | Ontario | Morris (2015) |
| Tho-012 mDUP | deer *(Odocoileus virginianus)* | 5.87 | Ontario | Morris (2015) |
| Tho-018 | deer *(Odocoileus virginianus)* | 5.69 | Ontario | Morris (2015) |
| Van-001 | deer *(Odocoileus virginianus)* | 5.52 | Ontario | Morris (2015) |
| Van-001 mDUP | deer *(Odocoileus virginianus)* | 5.82 | Ontario | Morris (2015) |
| Van-003 | deer *(Odocoileus virginianus)* | 4.49 | Ontario | Morris (2015) |
| Van003 mDUP | deer *(Odocoileus virginianus)* | 4.47 | Ontario | Morris (2015) |
| Van-018 | deer *(Odocoileus virginianus)* | 6.33 | Ontario | Morris (2015) |
| Van-019 | deer *(Odocoileus virginianus)* | 5.56 | Ontario | Morris (2015) |
| Van-020 | deer *(Odocoileus virginianus)* | 5.35 | Ontario | Morris (2015) |
| Van-022 | deer *(Odocoileus virginianus)* | 4.92 | Ontario | Morris (2015) |
| Van-108 | deer *(Odocoileus virginianus)* | 5.45 | Ontario | Morris (2015) |
| Wal-003 | deer *(Odocoileus virginianus)* | 4.18 | Ontario | Morris (2015) |
| Wal-005 | deer *(Odocoileus virginianus)* | 4.29 | Ontario | Morris (2015) |
| Wal-008 | deer *(Odocoileus virginianus)* | 6.11 | Ontario | Morris (2015) |
| Wal-009 | deer *(Odocoileus virginianus)* | 5.86 | Ontario | Morris (2015) |
| Wal-010 | deer *(Odocoileus virginianus)* | 5.54 | Ontario | Morris (2015) |
| Wal-011 | deer *(Odocoileus virginianus)* | 4.77 | Ontario | Morris (2015) |
| Wal-013 | deer *(Odocoileus virginianus)* | 4.49 | Ontario | Morris (2015) |
| Wal-014 | deer *(Odocoileus virginianus)* | 5.61 | Ontario | Morris (2015) |
| Wal-014 | deer *(Odocoileus virginianus)* | 4.26 | Ontario | Morris (2015) |
| Wal-016 | deer *(Odocoileus virginianus)* | 4.26 | Ontario | Morris (2015) |
| Wal-018 | deer *(Odocoileus virginianus)* | 5.08 | Ontario | Morris (2015) |
| Wal-021 | deer *(Odocoileus virginianus)* | 5.35 | Ontario | Morris (2015) |
| Wal-036 | deer *(Odocoileus virginianus)* | 6.13 | Ontario | Morris (2015) |
| Wal-037 | deer *(Odocoileus virginianus)* | 4.76 | Ontario | Morris (2015) |
| Wal-038 | deer *(Odocoileus virginianus)* | 5.54 | Ontario | Morris (2015) |
| Win-157 | deer *(Odocoileus virginianus)* | 8.17 | Ontario | Morris (2015) |
| Win-159 | deer *(Odocoileus virginianus)* | 7.29 | Ontario | Morris (2015) |
| BrB-02 | deer *(Odocoileus virginianus)* | 5.5 | Ontario | Morris (2015) |
| BrB-03 | deer *(Odocoileus virginianus)* | 5.28 | Ontario | Morris (2015) |
| Clv-033 + | deer *(Odocoileus virginianus)* | 6.25 | Ontario | Morris (2015) |
| Crf-043~ | deer *(Odocoileus virginianus)* | 6.04 | Ontario | Morris (2015) |
| Crf-044~ | deer *(Odocoileus virginianus)* | 5.77 | Ontario | Morris (2015) |
| Crf-045~ | deer *(Odocoileus virginianus)* | 6.74 | Ontario | Morris (2015) |
| Crf-046~ | deer *(Odocoileus virginianus)* | 6.6 | Ontario | Morris (2015) |
| Crf-047~ | deer *(Odocoileus virginianus)* | 7.24 | Ontario | Morris (2015) |
| Crf-048~ | deer *(Odocoileus virginianus)* | 6.56 | Ontario | Morris (2015) |
| Crf-051~ | deer *(Odocoileus virginianus)* | 6.17 | Ontario | Morris (2015) |
| Fon-020 | deer *(Odocoileus virginianus)* | 6.31 | Ontario | Morris (2015) |
| Fon-033 | deer *(Odocoileus virginianus)* | 6.9 | Ontario | Morris (2015) |
| Fon-104 | deer *(Odocoileus virginianus)* | 5.39 | Ontario | Morris (2015) |
| Ham-05 | deer *(Odocoileus virginianus)* | 8.17 | Ontario | Morris (2015) |
| Ham-06 | deer *(Odocoileus virginianus)* | 6.25 | Ontario | Morris (2015) |
| Ham-07 | deer *(Odocoileus virginianus)* | 6.13 | Ontario | Morris (2015) |
| Ham-08 | deer *(Odocoileus virginianus)* | 6.75 | Ontario | Morris (2015) |
| Ham-09~ | deer *(Odocoileus virginianus)* | 6.39 | Ontario | Morris (2015) |
| Ham-10~ | deer *(Odocoileus virginianus)* | 4.88 | Ontario | Morris (2015) |
| Ham-11~ | deer *(Odocoileus virginianus)* | 5.55 | Ontario | Morris (2015) |
| IWP(01)-30 | deer *(Odocoileus virginianus)* | 6.75 | Ontario | Morris (2015) |
| IWP(01)-30 DUP | deer *(Odocoileus virginianus)* | 6.76 | Ontario | Morris (2015) |
| IWP(03)-02 | deer *(Odocoileus virginianus)* | 6.76 | Ontario | Morris (2015) |
| IWP(03)-06 | deer *(Odocoileus virginianus)* | 6.58 | Ontario | Morris (2015) |
| IWP(03)-07 | deer *(Odocoileus virginianus)* | 5.79 | Ontario | Morris (2015) |
| IWP(03)-08 | deer *(Odocoileus virginianus)* | 5.47 | Ontario | Morris (2015) |
| IWP(03)-15 | deer *(Odocoileus virginianus)* | 6.14 | Ontario | Morris (2015) |
| IWP(09)-009 | deer *(Odocoileus virginianus)* | 5.67 | Ontario | Morris (2015) |
| IWP(09)-012 | deer *(Odocoileus virginianus)* | 5.75 | Ontario | Morris (2015) |
| IWP(09)-012 DUP | deer *(Odocoileus virginianus)* | 6.6 | Ontario | Morris (2015) |
| IWP(09)-032 | deer *(Odocoileus virginianus)* | 6.78 | Ontario | Morris (2015) |
| IWP(09)-048 | deer *(Odocoileus virginianus)* | 4.72 | Ontario | Morris (2015) |
| IWP(09)-079 | deer *(Odocoileus virginianus)* | 6.67 | Ontario | Morris (2015) |
| IWP(09)-083 | deer *(Odocoileus virginianus)* | 5.96 | Ontario | Morris (2015) |
| IWP(09)-083 mDUP | deer *(Odocoileus virginianus)* | 6.29 | Ontario | Morris (2015) |
| IWP(09)-088 | deer *(Odocoileus virginianus)* | 8.49 | Ontario | Morris (2015) |
| IWP(09)-119 | deer *(Odocoileus virginianus)* | 5.71 | Ontario | Morris (2015) |
| IWP(09)-122 | deer *(Odocoileus virginianus)* | 7.64 | Ontario | Morris (2015) |
| Pip(1)-010^ | deer *(Odocoileus virginianus)* | 6.18 | Ontario | Morris (2015) |
| Pip(1)-023 + | deer *(Odocoileus virginianus)* | 6.39 | Ontario | Morris (2015) |
| Pip(1)-024 + | deer *(Odocoileus virginianus)* | 6.03 | Ontario | Morris (2015) |
| Pip(1)-024 mDUP | deer *(Odocoileus virginianus)* | 6.15 | Ontario | Morris (2015) |
| Pip(1)-024 mDUP DUP | deer *(Odocoileus virginianus)* | 5.21 | Ontario | Morris (2015) |
| Pip(1)-025 | deer *(Odocoileus virginianus)* | 6.03 | Ontario | Morris (2015) |
| Pip(1)-048 | deer *(Odocoileus virginianus)* | 6.84 | Ontario | Morris (2015) |
| Pip(1)-075 | deer *(Odocoileus virginianus)* | 8.49 | Ontario | Morris (2015) |
| Pip(1)-179 | deer *(Odocoileus virginianus)* | 5.97 | Ontario | Morris (2015) |
| Pip(1)-184 | deer *(Odocoileus virginianus)* | 5.44 | Ontario | Morris (2015) |
| Pip(2)-070 | deer *(Odocoileus virginianus)* | 6.86 | Ontario | Morris (2015) |
| Pri-007 | deer *(Odocoileus virginianus)* | 5.24 | Ontario | Morris (2015) |
| Rif-062 | deer *(Odocoileus virginianus)* | 7.12 | Ontario | Morris (2015) |
| Rif-080 | deer *(Odocoileus virginianus)* | 6.81 | Ontario | Morris (2015) |
| Rif-092 | deer *(Odocoileus virginianus)* | 6.78 | Ontario | Morris (2015) |
| Rif-092 mDUP | deer *(Odocoileus virginianus)* | 6.72 | Ontario | Morris (2015) |
| Rif-107 | deer *(Odocoileus virginianus)* | 5.18 | Ontario | Morris (2015) |
| Rif-107 DUP | deer *(Odocoileus virginianus)* | 5.05 | Ontario | Morris (2015) |
| Tho-035 | deer *(Odocoileus virginianus)* | 5.03 | Ontario | Morris (2015) |
| Tho-046 | deer *(Odocoileus virginianus)* | 5.86 | Ontario | Morris (2015) |
| Tho-054 | deer *(Odocoileus virginianus)* | 4 | Ontario | Morris (2015) |
| Tho-054 DUP | deer *(Odocoileus virginianus)* | 4.81 | Ontario | Morris (2015) |
| Tho-058 | deer *(Odocoileus virginianus)* | 4.87 | Ontario | Morris (2015) |
| Tho-065 | deer *(Odocoileus virginianus)* | 6.6 | Ontario | Morris (2015) |
| Van-011 | deer *(Odocoileus virginianus)* | 5.56 | Ontario | Morris (2015) |
| Van-012 | deer *(Odocoileus virginianus)* | 6.76 | Ontario | Morris (2015) |
| Van-017 | deer *(Odocoileus virginianus)* | 6.31 | Ontario | Morris (2015) |
| Wal-050 | deer *(Odocoileus virginianus)* | 5.46 | Ontario | Morris (2015) |
| Wal-050 mDUP | deer *(Odocoileus virginianus)* | 5.61 | Ontario | Morris (2015) |
| Wal-051 | deer *(Odocoileus virginianus)* | 5.63 | Ontario | Morris (2015) |
| Win-047 | deer *(Odocoileus virginianus)* | 6.92 | Ontario | Morris (2015) |
| Win-047 DUP | deer *(Odocoileus virginianus)* | 6.85 | Ontario | Morris (2015) |
| Win-221 | deer *(Odocoileus virginianus)* | 6.11 | Ontario | Morris (2015) |
| UCIAMS-204719 | deer (*Odocoileus virginianus*) | 5.00 | Northern NY | Abel et al. 2019 |
| UCIAMS-199806 | deer (*Odocoileus virginianus*) | 7.50 | Northern NY | Abel et al. 2019 |
| UCIAMS-204718 | deer (*Odocoileus virginianus*) | 6.40 | Northern NY | Abel et al. 2019 |
| UCIAMS-199805 | deer (*Odocoileus virginianus*) | 5.70 | Northern NY | Abel et al. 2019 |
| UCIAMS-199802 | deer (*Odocoileus virginianus*) | 6.80 | Northern NY | Abel et al. 2019 |
| UCIAMS-204714 | deer (*Odocoileus virginianus*) | 5.70 | Northern NY | Abel et al. 2019 |
| UCIAMS-204716 | deer (*Odocoileus virginianus*) | 5.70 | Northern NY | Abel et al. 2019 |
| UCIAMS-204722 | deer (*Odocoileus virginianus*) | 6.20 | Northern NY | Abel et al. 2019 |
| UCIAMS-204720 | deer (*Odocoileus virginianus*) | 5.80 | Northern NY | Abel et al. 2019 |
| UCIAMS-190554 | deer (*Odocoileus virginianus*) | 4.40 | Mohawk Valley | Manning and Hart 2019 |
| UCIAMS-190552 | deer (*Odocoileus virginianus*) | 4.50 | Mohawk Valley | Manning and Hart 2019 |
| UCIAMS-190553 | deer (*Odocoileus virginianus*) | 4.80 | Mohawk Valley | Manning and Hart 2019 |
| UCIAMS-192976 | deer (*Odocoileus virginianus*) | 8.40 | Mohawk Valley | Manning and Hart 2019 |
| UCIAMS-190557 | deer (*Odocoileus virginianus*) | 4.90 | Mohawk Valley | Manning and Hart 2019 |
| UCIAMS-192975 | deer (*Odocoileus virginianus*) | 4.60 | Mohawk Valley | Manning and Hart 2019 |
| UCIAMS-190555 | deer (*Odocoileus virginianus*) | 5.00 | Mohawk Valley | Manning and Hart 2019 |
| UCIAMS-190556 | deer (*Odocoileus virginianus*) | 4.60 | Mohawk Valley | Manning and Hart 2019 |
| UCIAMS-190559 | deer (*Odocoileus virginianus*) | 3.80 | Mohawk Valley | Manning and Hart 2019 |
| UCIAMS-190562 | deer (*Odocoileus virginianus*) | 4.30 | Mohawk Valley | Manning and Hart 2019 |
| UCIAMS-190561 | deer (*Odocoileus virginianus*) | 4.50 | Mohawk Valley | Manning and Hart 2019 |
| UCIAMS-190560 | deer (*Odocoileus virginianus*) | 4.70 | Mohawk Valley | Manning and Hart 2019 |
| UCIAMS-190551 | deer (*Odocoileus virginianus*) | 4.40 | Mohawk Valley | Manning and Hart 2019 |
| UCIAMS-190549 | deer (*Odocoileus virginianus*) | 4.40 | Mohawk Valley | Manning and Hart 2019 |
| UCIAMS-190550 | deer (*Odocoileus virginianus*) | 5.00 | Mohawk Valley | Manning and Hart 2019 |
| UCIAMS-190548 | deer (*Odocoileus virginianus*) | 3.90 | Mohawk Valley | Manning and Hart 2019 |
| UCIAMS-190546 | deer (*Odocoileus virginianus*) | 5.1 | Mohawk Valley | Manning and Hart 2019 |
| UCIAMS-190547 | deer (*Odocoileus virginianus*) | 5.00 | Mohawk Valley | Manning and Hart 2019 |
| UCIAMS-190566 | deer (*Odocoileus virginianus*) | 4.70 | Mohawk Valley | Manning and Hart 2019 |
| UCIAMS-190565 | deer (*Odocoileus virginianus*) | 5.90 | Mohawk Valley | Manning and Hart 2019 |
| UCIAMS-190563 | deer (*Odocoileus virginianus*) | 5.00 | Mohawk Valley | Manning and Hart 2019 |
| UCIAMS-190564 | deer (*Odocoileus virginianus*) | 3.70 | Mohawk Valley | Manning and Hart 2019 |
| UCIAMS190544 | deer (*Odocoileus virginianus*) | 4.10 | Mohawk Valley | Manning and Hart 2019 |
| UCIAMS190542 | deer (*Odocoileus virginianus*) | 4.20 | Mohawk Valley | Manning and Hart 2019 |
| UCIAMS190543 | deer (*Odocoileus virginianus*) | 4.00 | Mohawk Valley | Manning and Hart 2019 |
| UCIAMS-192977 | deer (*Odocoileus virginianus*) | 4.40 | Mohawk Valley | Manning and Hart 2019 |
| Cra-015 | groundhog *(Marmota monax)* | 4.25 | Ontario | Morris (2015) |
| Fon-025 | groundhog *(Marmota monax)* | 2.53 | Ontario | Morris (2015) |
| Fon-049 | groundhog *(Marmota monax)* | 3.94 | Ontario | Morris (2015) |
| Lig-004 | groundhog *(Marmota monax)* | 2.27 | Ontario | Morris (2015) |
| Lig-009 | groundhog *(Marmota monax)* | 2.79 | Ontario | Morris (2015) |
| Lig-014 | groundhog *(Marmota monax)* | 2.67 | Ontario | Morris (2015) |
| Tho-007 | groundhog *(Marmota monax)* | 2.27 | Ontario | Morris (2015) |
| Van-044 | groundhog *(Marmota monax)* | 3.09 | Ontario | Morris (2015) |
| Van-056 | groundhog *(Marmota monax)* | 3.11 | Ontario | Morris (2015) |
| Van-069 | groundhog *(Marmota monax)* | 3.05 | Ontario | Morris (2015) |
| Van-072 | groundhog *(Marmota monax)* | 3.13 | Ontario | Morris (2015) |
| Van-080 | groundhog *(Marmota monax)* | 2.73 | Ontario | Morris (2015) |
| Van-093 | groundhog *(Marmota monax)* | 3.2 | Ontario | Morris (2015) |
| Van-095 | groundhog *(Marmota monax)* | 2.95 | Ontario | Morris (2015) |
| Van-113 | groundhog *(Marmota monax)* | 4.86 | Ontario | Morris (2015) |
| Van-119 | groundhog *(Marmota monax)* | 3.01 | Ontario | Morris (2015) |
| Wal-017 | groundhog *(Marmota monax)* | 2.13 | Ontario | Morris (2015) |
| Wal-020 | groundhog *(Marmota monax)* | 3.34 | Ontario | Morris (2015) |
| OLG-015 | rabbit (Leporidae) | 4.66 | Ontario | Morris (2015) |
| Pip(2)-017 | rabbit (Leporidae) | 4.11 | Ontario | Morris (2015) |
| Tho-019 | rabbit (Leporidae) | 3.44 | Ontario | Morris (2015) |
| Tho-023 | rabbit (Leporidae) | 3.46 | Ontario | Morris (2015) |
| Van-068 | rabbit (Leporidae) | 3.96 | Ontario | Morris (2015) |
| Van-118 | rabbit (Leporidae) | 4.14 | Ontario | Morris (2015) |
| Wal-024 | rabbit (Leporidae) | 4.08 | Ontario | Morris (2015) |
| Wal-053 | rabbit (Leporidae) | 2.09 | Ontario | Morris (2015) |

Sources:

Abel, Timothy J., Jessica Vavrasek, and John P. Hart. 2019. Radiocarbon Dating the Iroquoian Occupation of Northern New York. American Antiquity 84. <https://dx.doi/10.1017/aaq.2019.50>

Booth, L. 2014 *A Stable Isotope Analysis of Faunal Remains from Special Deposits on Ontario Iroquoian Tradition Sites* (The University of Western Ontario). <https://ir.lib.uwo.ca/etd/2644>

Manning, S, Hart, JP. 2019. Radiocarbon, Bayesian chronological modeling and early European metal circulation in the sixteenth-century AD Mohawk River Valley, USA. Submitted

Morris, Z. H. 2015 *Reconstructing Subsistence Practices of Southwestern Ontario Late Woodland Peoples (A.D. 900-1600) Using Stable Isotopic Analyses of Faunal Material* (The University of Western Ontario). <https://ir.lib.uwo.ca/etd/2921>
