# Supplementary material for: Using Maize δ15N values to assess soil fertility in fifteenth- and sixteenth-century ad Iroquoian agricultural fields
Source: PLoS One. 2020 Apr 8;15(4):e0230952. doi: 10.1371/journal.pone.0230952 (PMC7141618; doi:10.1371/journal.pone.0230952)
Supplement: Attachment — Submitted filename: Review of PONE-D-19-26845.docx [file pone.0230952.s003.docx]

Review of PONE-D-19-26845

Title: *Using Maize δ15N Values to Assess Soil Fertility in Fifteenth- and Sixteenth-Century AD Iroquoian Agricultural Fields*

Authors: *John P. Hart and Robert S. Ferenec*

***General Comments to Editor and Authors:***

This paper provides a compelling case for Iroquoian farmers in New York State, USA and Ontario and Québec in Canada having maintained the fertility of their agricultural fields during the 15^th^ and 16^th^ centuries AD. Specifically, the authors obtained δ^15^N and δ^13^C values on maize kernels from >60 Iroquoian archaeological sites, and performed statistical analysis on these isotopic data, to elucidate upon the fertility of the soils that once supported maize at these sites. The authors analyzed these isotopes in the absence of preserved Iroquoian fields, therefore, δ^15^N and δ^13^C values on maize kernels can be viewed as an indirect proxy for soil productivity (an idea not fully fleshed out in the manuscript, see below). To strengthen the hypothesis that Iroquoian farmers burned their maize fields after harvest to input plant-available nitrogen, suggested from analysis of the archaeological material, the authors conducted an experimental study. The authors charred and performed isotopic analysis upon modern maize kernels as well as grass seeds collected in the 1930s-1940s, which validated their interpretations of the isotopic data based on Iroquoian archaeological material. The authors’ results were also compared to the experimental data on wheat kernels derived from archaeological sites in the Eastern Hemisphere.

The key finding of this research, presented for the first time in this paper (to my knowledge), is that the amount of nitrogen input into the soils through burning by Iroquoian farmers during the 15^th^ and 16^th^ centuries AD was comparable to adding manure to agricultural fields by Neolithic farmers in Europe. This finding is highly significant and requires to be more strongly highlighted in the paper (below I indicate where in the manuscript). Additionally, I recommend that the authors expound upon other aspects of their findings and the broader significance of their research in their revision of this manuscript.

In my opinion, **this manuscript is worthy of publication in *PLOS ONE* pending minor revisions**. The paper describes original research not published elsewhere. The theoretical and methodological approaches and explanations are robust and the statistical analyses are excellent; for example, the reasoning expressed in the paragraph on P. 7, Lns 188-205, is exceptional. The conclusions are well supported by the data. The research adheres to integrity standards, and the data will be available upon publication. The paper is also well written. Below are more specific comments to aid the authors when revising this paper.

*Specific Comments (my suggested textual additions are in bolded red font):*

| Page # | Line # | Comments:  * My textual additions are in red font. |
| --- | --- | --- |
| 1 | 6, 32 | Abstract & Introduction: insert “State, USA” after New York, and “Canada” after “Québec” [use the letter e with an acute accent, which is the official spelling of this province’s name], to read “New York State, USA and Ontario and Québec, Canada,”. |
| 1 | 9-12 | The authors did not mention an experimental component of their research, where they charred modern maize kernels and 20^th^ century grass seeds to valid their isotopic values obtained from maize kernels obtained from archaeological results. Therefore, a short sentence regarding this work should be added to the Abstract. |
| 1 | 13 | Abstract: add a phrase to the end of this sentence, or an additional sentence, that identifies the broader significance of this research beyond Iroquoian studies. Here is a suggestion: “These results…Iroquoian agronomic practices, and identified that Iroquoian farmers augmented nitrogen through burning, producing values comparable to European Neolithic farmers who utilized manure to upkeep soil productivity.” |
| 1 | 46 | Provide the source for this sentence about 3-4 maize plants per mound. |
| 3 | 98-99 | Write a sentence or two that explains why there is an “apparent current lack of Iroquoian agricultural field preservation.” I suggest that the authors move up the first sentence of the following paragraph (Ln 116-117) about depletion of soil organic matter to Ln 98. Also, add a statement that the areas where Iroquoian agriculture fields were once located have been heavily farmed by Euro-Americans and Euro-Canadians in subsequent decades, thereby obliterating the soil characteristics of the fields farmed centuries ago by indigenous populations. |
| 3 | 101-105 | Please divide this long sentence into two. |
| 3 | 113 | Insert “directly from the mounds themselves” between “evidence” and “to test,” given that the paper does provide archaeological evidence to test this hypothesis. |
| 3 | 116 | Insert an introductory sentence, before the one beginning with “Eastern Hemisphere grains…”, which explains that in the absence of preserved Iroquoian agriculture fields one can use δ^15^N values obtained from cultigens as an indirect proxy for past soil productivity. |
| 6 | 152 | Table 2: at the bottom of this table, provide footnotes with details, including the full names, pertaining to the two labs used, UGAMS and UCIAMS (which are identified on p. 12). Note: I approve of the authors using only two labs to obtain these δ^15^N and δ^13^C data, to minimize any possible data discrepancy between them. While looking at the data in this table, I did not see any indication of variation in the results produced between the two labs, as both labs gave a similar range of values for each isotope. Additionally, these AMS labs have reputations for producing outstanding (reliable) results. |
| 6 | 155 | The colored dots are not identified in Fig. 1. Therefore, the authors should insert a legend explaining the color scheme for these dots, which indicate site locations in southern Ontario, northern New York, and the Mohawk Valley. This information could be added to the figure caption, but a better option is to embed a legend in this figure. |
| 6 | 161 | Identify the grain as being wheat (*Triticum* spp.). |
| 6 | 173-175 | Move this sentence starting with “The kernels heated at 220°C fully carbonized…” to integrate with the sentence starting on Ln 177, “Those heated to 220°C…” to reduce redundancy. |
| 7, 9 | 201, 250 | Is “cal” necessary, given that BC/AD are used? |
| 7 | 211 | Mention the three subregions at the end of this caption for Fig. 2 (d). |
| 8 | 213 | Replace Quebec with Québec. |
| 10 | 285 | Delete “in” before “can.” |
| 11 | 315 | Insert “involving fire” after agronomic practices. |
