# Supplementary material for: Using Maize δ15N values to assess soil fertility in fifteenth- and sixteenth-century ad Iroquoian agricultural fields
Source: PLoS One. 2020 Apr 8;15(4):e0230952. doi: 10.1371/journal.pone.0230952 (PMC7141618; doi:10.1371/journal.pone.0230952)
Supplement: Attachment — Submitted filename: Response to reviewers.docx [file pone.0230952.s004.docx]

Responses to Reviewers

Reviewer 1

I appreciate your effort do use d15N values to assess the ancient agriculture practices. Many

are the examples of ancient agriculture that was able to support dense populations (Asia) using

a variety of soil amendments (in the obvious absence of fertilizers). However, authors might

use a more precise interpretation of d15N values to draw their conclusions. Nitrogen isotope

natural abundance (expressed here as d15N values) is not an indicator to “plant- available

nitrogen levels”. Thus, the assumption of authors that the higher the d15N values (plant), the

higher the plant-available nitrogen levels” is not correct. I fact, the N transformation processes

lead to fractionation of nitrogen isotope and the N loss (in soli or manure storage) results in an

increasing of d15N values. Manures have high d15N values manly due to N loss as ammonia

(volatilization). Plants tend to mirror the d15N values of available N in soil which can reflect

the N source, i.e. manure and plant residues and biological N fixing most of the time (I am

excluding NH3 deposition for this case). Thus, high d15N indicates loss of N from the system

(i.e. soil). However, soil amended with animal manure tends to show high d15N values. I

recommend to the authors to bring to support their discussion two good reviews: CHOI, W. J.

et al. Synthetic fertilizer and livestock manure differently affect δ15N in the agricultural

landscape: A review. Agriculture, Ecosystems & Environment, v. 237, p. 1-15, 2017, and

Martinelli LA, Piccolo MC, Townsend AR, Vitousek PM, Cuevas E, McDowell W et al.,

Nitrogen stable isotopic composition of leaves and soil: tropical versus temperate forests.

Biogeochemistry 46:45–65 (1999). The last shows d15N values of soil and leaves (temperate

vs. tropical forests). Tropical forests have higher d15N values than Temperate Forests.

Despite of my opinion that the authors might refine the discussion using the right

interpretation of d15N values as above mentioned, they are right suggesting a more relevant

role of N-fixing from common bean instead use of animal manures based on d15N values

obtained. But at this point the paper also needs to give more information about the d15N of N fixing plants (ranges, common values, etc). This is crucial to support their right conclusions.

Thus, I recommend the publication of this manuscript after revision.

*Thank you for your comments. We have addressed this issue through the addition of line 125 through 129.*

Reviewer 2

1 6, 32 Abstract & Introduction: insert “State, USA” after New York, and “Canada” after

“Québec” [use the letter e with an acute accent, which is the official spelling of this province’s

name], to read “New York State, USA and Ontario and Québec, Canada,”.

*We have made these modifications except adding State after New York.*

1 9-12 The authors did not mention an experimental component of their research, where they

charred modern maize kernels and 20th century grass seeds to valid their isotopic values

obtained from maize kernels obtained from archaeological results. Therefore, a short sentence

regarding this work should be added to the Abstract.

*Done*

1 13 Abstract: add a phrase to the end of this sentence, or an additional sentence, that

identifies the broader significance of this research beyond Iroquoian studies. Here is a

suggestion: “These results…Iroquoian agronomic practices, and identified that Iroquoian

farmers augmented nitrogen through burning, producing values comparable to European

Neolithic farmers who utilized manure to upkeep soil productivity.”

*Done*

1 46 Provide the source for this sentence about 3-4 maize plants per mound.

*Done*

3 98-99 Write a sentence or two that explains why there is an “apparent current lack of

Iroquoian agricultural field preservation.” I suggest that the authors move up the first sentence

of the following paragraph (Ln 116-117) about depletion of soil organic matter to Ln 98. Also,

add a statement that the areas where Iroquoian agriculture fields were once located have been

heavily farmed by Euro-Americans and Euro-Canadians in subsequent decades, thereby

obliterating the soil characteristics of the fields farmed centuries ago by indigenous

populations.

*Done*

3 101-105 Please divide this long sentence into two.

*Done*

3 113 Insert “directly from the mounds themselves” between “evidence” and “to test,” given

that the paper does provide archaeological evidence to test this hypothesis.

*Done*

3 116 Insert an introductory sentence, before the one beginning with “Eastern Hemisphere

grains…”, which explains that in the absence of preserved Iroquoian agriculture fields one can

use δ15N values obtained from cultigens as an indirect proxy for past soil productivity.

*We have chosen to keep the beginning of this paragraph as in the original.*

6 152 Table 2: at the bottom of this table, provide footnotes with details, including the full

names, pertaining to the two labs used, UGAMS and UCIAMS (which are identified on p. 12).

*Done*

6 155 The colored dots are not identified in Fig. 1. Therefore, the authors should insert a

legend explaining the color scheme for these dots, which indicate site locations in southern

Ontario, northern New York, and the Mohawk Valley. This information could be added to the

figure caption, but a better option is to embed a legend in this figure.

*There is now a key to the colors in the figure.*

6 161 Identify the grain as being wheat (Triticum spp.).

*Done*

6 173-175 Move this sentence starting with “The kernels heated at 220°C fully carbonized…”

to integrate with the sentence starting on Ln 177, “Those heated to 220°C…” to reduce

redundancy.

*We have rewritten this paragraph to clarify and avoid redundancy.*

7, 9 201, 250 Is “cal” necessary, given that BC/AD are used?

We have deleted the “cal”.

7 211 Mention the three subregions at the end of this caption for Fig. 2 (d).

We have added a key to the dot colors.

8 213 Replace Quebec with Québec.

Done

10 285 Delete “in” before “can.”

*We eliminated this table after receiving additional low values on archeological maize, which serve as a better baseline for understanding the elevated values on archaeological maize.*

11 315 Insert “involving fire” after agronomic practices.

*Because this is only one possible aspect of the agronomic practices be have declided to modify this sentence.*
